# Potential application of miRNAs as diagnostic and therapeutic tools in chronic pancreatitis

**DOI:** 10.1111/jcmm.12603

**Published:** 2015-07-06

**Authors:** Liang-Hao Hu, Jun-Tao Ji, Zhao-Shen Li

**Affiliations:** Department of Gastroenterology, Changhai Hospital, The Second Military Medical UniversityShanghai, China

**Keywords:** chronic pancreatitis, miRNA, diagnosis, therapeutic tools, fibrosis

## Abstract

Chronic pancreatitis (CP) is a progressive inflammatory disease typified by end-stage fibrosis. This disease can also increase the risk of pancreatic cancer. The associated diagnosis, pain and other complications further add to the burden of disease management. In recent years, significant progress has been achieved in identifying miRNAs and their physiological functions, including mRNA repression and protein expression control. Given the extensive effort made on miRNA research, a close correlation has been discovered between certain types of miRNAs and disease progression, particularly for tissue fibrosis. Designing miRNA-related tools for disease diagnosis and therapeutic treatments presents a novel and potential research frontier. In the current review, we discuss various miRNAs closely interacting with CP, as well as the possible development of targeted miRNA therapies in managing this disease.

## Introduction

Chronic pancreatitis (CP) is a progressive fibro-inflammatory disease or chronic inflammation of the pancreas characterized by calcifications, necrosis, fatty replacement, fibrosis and other pathophysiological responses 1–3. Chronic abdominal pain, pancreatic steatorrhea and elevated blood glucose level are the major clinical characteristics of CP. Many risk factors are associated with CP progression. Chronic pancreatitis results from a complex combination of environmental (*e.g*. alcohol, cigarettes and occupational chemicals) and genetic factors [*e.g*. mutation in a trypsin-controlling gene or cystic fibrosis transmembrane conductance regulator (CFTR)]. Currently, the major clinical diagnostic tools are computed tomography, magnetic resonance imaging and endoscopic ultrasound. Although these imaging assessments are valuable and mostly practical, these procedures are expensive and invasive. Other clinical and laboratory investigations also comprise integral parts of CP diagnosis. However, diagnosis becomes complicated when patients come for clinicians after acute inflammation has already occurred. Chronic pancreatitis management usually consists of combined treatments of pancreatic enzyme replacement therapy, pain treatments 4, endotherapies 5–7, extracorporeal shock wave lithotripsy and surgeries 8–11. Unfortunately, these therapies are rarely effective against fibrosis. Recently, delivery of VA-lip-siRNAgp46 in fibrotic cells has reversed pancreatic fibrosis in rats 12. However, more targets for new drugs specifically designed as antifibrotics are required. Simultaneously, the development of non-invasive fibrogenic markers, as well as combined scoring systems incorporating serum and clinical features, will improve assessment of therapeutic response. Considering the close link between miRNAs and fibrosis, miRNAs may offer novel important diagnostic and therapeutic tools to inhibit the progress of CP. In this review, we will outline the potential of miRNAs in interfering with CP.

miRNAs, a class of non-coding RNAs, modulate the expression of various genes and currently appear as potential therapeutic targets to interfere with the genetic factors complicating CP. One of the major actions of miRNAs is the post-transcriptional repression of the synthesis of their targets. Nucleotides 2–8 at the 5′ end of a mature miRNA are referred to as the ‘seed sequences’ essential for miRNA-mediated gene silencing 13. Recent studies also revealed the important role of miRNAs in modulating the expression of various cytokines and chemokines 14, which participate in inflammation infiltration and complication of multiple diseases, including pancreatic fibrosis and CP 15,16. Interestingly, aberrant miRNA expression can also be used as indicator for disease progression, which consequently offers a potential tool for diagnosis 17. Individual miRNAs engage numerous mRNA targets. Therefore, modulation of miRNA levels represents a significant disease intervention, which may be targeting multiple disease progression at one setting. The ability to therapeutically manipulate miRNA expression and to function through systemic or local delivery of miRNA inhibitors, referred to as anti-miRs, has elicited research interest for miRNAs as novel therapeutic targets 18. However, our current understanding about the roles of miRNAs in CP regulation remains limited. In this review, we summarize the interlinked relations between miRNAs and CP, specifically for pancreatic fibrosis, to offer a potential diagnostic and therapeutic tool in CP treatment.

## Pathology of chronic pancreatitis

**Figure 1:**
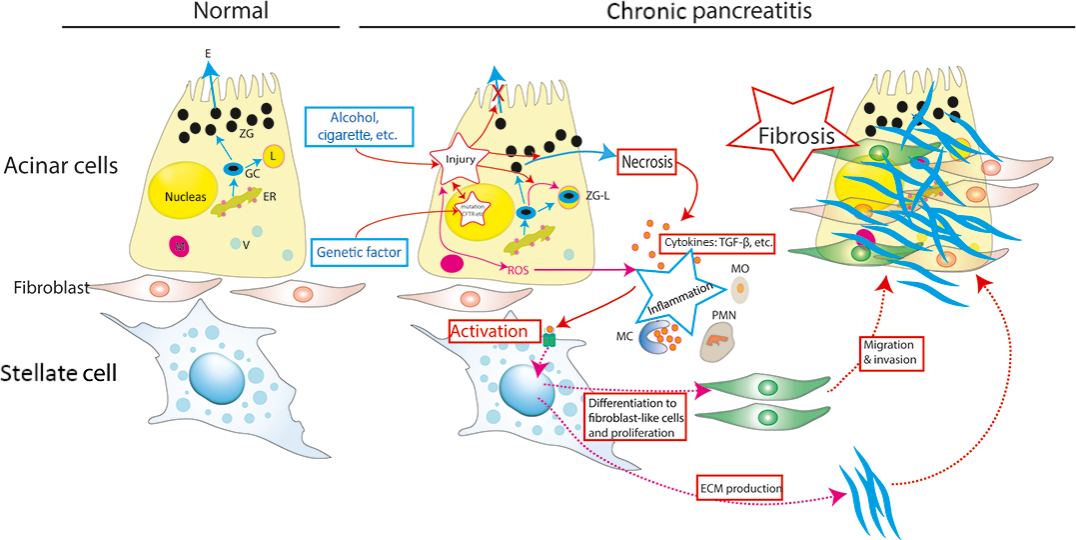
Current concept of the pathogenesis of chronic pancreatitis. An acinar cell is susceptible to autodigestive injury for both genetic and environmental causes. In the presence of an appropriate trigger factor, overt acinar cell injury is initiated. These events result in inflammation. A pancreatic stellate cell (PSC) is activated by cytokines released during pancreatic inflammation, leading to excessive ECM production. The PSCs then proliferate, migrate, invade, and differentiate to fibroblast-like cells. Activation of stellate cells is increased by cytokines from infiltrating leucocytes and the injured acinar cell; thus, fibrosis is shown. The end stage of chronic pancreatitis is identified by loss of all secretory tissues, disappearance of inflammatory cells, and severe fibrosis. ER, endoplasmic reticulum; ZG, zymogen granules; GC, Golgi complex; E, trypsinogen, and other precursor proteases; ROS, reactive oxygen species, L, lysosomes; ZG-L, miniscule fraction of zymogens activated by co-localization with lysosomal enzymes; MC, mast cell; PMN, polymorphonuclear cell; MO, monocyte; V, Vesicle; and ECM, extracellular matrix protein.

Experimental studies since the 1950s have shown that an attack of pancreatitis begins as pancreastasis or the prevention of apical exocytosis in the pancreatic acinar cell 19. Experimental work has indicated a burst of reactive oxygen species (ROS) that triggered the so-called ‘pancreastasis’ 20; the ROS is regarded as the potentiator of inflammation, which activates signalling cascades by converting the damaged acinar cell into a factory for chemokines and cytokines. Fibrosis indicates that interstitial stellate cells are activated in CP; these cells play a central part in disease progression by regulating the synthesis and degradation of extracellular matrix (ECM) proteins 21. Histochemical findings suggest a causal influence of two factors: an increase in lipid peroxidation products caused by an excess of ROS in adjacent acinar cells 22 and the release of mast cell degranulation products 23, particularly transforming growth factor-β1 (TGF-β1) 24. These two factors are linked because the ROS and their oxidation products are natural activators of mast cells 1. Activation of pancreatic stellate cells (PSCs) is increased by cytokines from infiltrating leucocytes and injured acinar cells 25. The activated PSCs secrete excessive amounts of ECM proteins comprising fibrous tissue. Consequently, the end stage of CP is characterized by loss of all secretory tissues, disappearance of inflammatory cells and severe fibrosis (Fig.1). Nevertheless, given insufficient evidence, no consensus exists regarding the mechanisms by which these diverse causative factors lead to CP 1.

Pancreatic fibrosis is an irreversible lesion that can disrupt pancreatic exocrine and endocrine functions. To efficiently manage CP, processes leading to tissue fibrosis should be investigated. At present, PSCs are established as key players in pancreatic fibrogenesis. Reducing inflammation infiltration through cytokine and chemokine production, and halting the process of genetic or external regulated activation of PSCs can both serve as potential pathways. Pancreatic stellate cells are comparable to hepatic stellate cells (HSCs) because they share similar morphology and functions. Studies on the role of HSCs during fibrosis are extensive and suggest that divergent miRNAs participate in the liver fibrotic process and activation of HSCs 26. Therefore, miRNAs may play an important role in the development of pancreatic fibrosis because of their diverse functions, including the ability to modulate cytokine expression. Hence, we consider miRNAs as potential efficacious treatment targets for CP.

## Aberrant miRNA expression patterns in chronic pancreatitis

In humans, aberrant expression of miRNAs contributes to the production of atypical levels of proteins, such as TGFs, which promote inflammatory infiltration during CP. Remarkably, as CP is closely linked to genetic mutation, miRNAs are also significantly involved in this process by promoting or inhibiting the expression of mRNAs of certain genes, which are discussed below, hence demonstrating a significant modulation of protein profiles 27. To date, limited research has provided evidence on the mechanism of miRNA interference with CP. However, many studies have been conducted on the relation between miRNAs and pancreatic cancer 28,29. Usually, researchers would compare pancreatic cancer tissues with CP and normal tissues. Bloomston, *et al*. 30 studied and compared the miRNA expression profiles between normal and CP patients. Their study collected RNA extracted from CP specimens and benign adjacent pancreatic tissues, which were then hybridized to miRNA microarrays. Significance and prediction analyses of microarrays were performed for identification. As a result, >10 miRNAs overexpressed in CP compared with normal tissues, and two miRNAs, namely, miR-96 and miR-497, were suppressed in CP tissues. These results provide evidence regarding the association between miRNAs and CP. Although further mechanism studies are needed to investigate whether the expression profiles of these miRNAs play significant roles in modulating disease progression, the aberrant expression profiles initially provided a source for disease diagnosis and offered potential therapeutic targets to inhibit disease progression.

Despite the absence of established links between CP and miRNAs, the complex links between CP and other diseases, such as pancreatic cancer 15,31, acute pancreatitis 17 and other liver diseases 26, render a potential aspect to study miRNA aberrant profiles in CP. Eight miRNAs were significantly up-regulated in most pancreatic cancer tissues and cell lines. These miRNAs were miR-196a, miR-190, miR-186, miR-221, miR-222, miR-200b, miR-15b and miR-95 28,32. Previous studies suggested that the aberrant miRNAs in serum or faeces can be adopted as novel biomarkers for pancreatic cancer 33,34. Pancreas-specific miRNAs, namely, miR-216a and miR-216b, are also significantly increased in serum of a rat model of acute pancreatitis 17,35. Identification of significant miRNAs specific to CP remains to be a frontier, but the existing abnormal expression profiles discussed may be the first ones to be studied. Previous research also indicated that the most common causes of CP are alcohol or idiopathic 1, and the TGF-β-activated PSCs are indispensable in CP progression. Therefore, we will discuss the relationship between miRNAs and the three parts of CP pathogenesis.

## miRNA and alcohol-induced CP

Alcohol is one of the independent disease initiators in CP. Although patients who have never consumed alcohol can develop pancreatitis, alcohol can increase the sensitivity of the pancreas to injuries from other risk factors. The prevalence is increased fourfold among participants with history of alcoholism compared with those without 36. Alcohol use is the single most common cause of CP 37,38.

Up-regulation of miR-203, miR-205 and miR-223 was identified in patients with regular alcohol consumption 36. Moreover, miR-34a is closely related to alcohol consumption. Meng *et al*. 39 found that miR-34a is up-regulated in ethanol-exposed mouse liver *in vivo* and contributes to alcoholic liver fibrosis and tissue repair by regulating cell proliferation, remodelling and migration. The increased cell survival and migration by miR-34a may be important for the regression of alcohol-induced liver fibrosis and cirrhosis, but may also modulate the high potential for malignant transformation.

Besides liver fibrosis, miR-34 can also modulate a wide range of tissue fibrosis and fibrogenesis. A possible mechanism is the modulation of miR-34a that alters the expression of matrix metalloproteases 1 and 2 (MMP-1, MMP-2), which are the mediators involved in cell remodelling during alcohol-induced fibrosis 40. Although few studies have been conducted regarding the correlation between miR-34a and alcohol-induced pancreatitis, miR-34a is possibly up-regulated by alcohol overconsumption, thereby leading to fibrosis in CP. Similarly, the miRNAs related to alcohol consumption may play important roles in pancreatic fibrosis. Furthermore, the miR-34a expression can be suppressed by elevated TGF-β activity in other diseases 41. Thus, miR-34a or other certain miRNAs may be used as potential biomarkers for CP, especially in patients with high alcohol consumption.

## miRNAs regulating CP-related gene expression

One of the major genes implicated in CP is CFTR, which encodes a protein involved in the transport of chloride ions across cell membranes 42. The mutation from CFTR gene results in cystic fibrosis 43. The predominant mechanisms controlling CFTR expression levels *in vivo* are driven by cis-acting regulatory elements 44. However, miRNAs can also play an important role in directing changes in the amounts of mature CFTR proteins, which can significantly influence the cell physiology. Inhibition (by antagomirs or anti-miRs) of miRNAs targeting CFTR in relevant tissues can elevate CFTR expression levels to within the normal range. The 3′-untranslated region (3′-UTR) of CFTR mRNA, the putative site of miRNA annealing, is highly conserved in different species and contains several regulatory motifs important for the mRNA stability and translation control 45. Selective miRNAs, such as miR-145, miR-494 and miR-101, as discussed below, exhibit similar 3′-UTRs to pair for positive interactions 13. This pairing results in targeted and functional suppression of CFTR mRNA translation, thereby leading to post-transcriptional regulation of CFTR.

Megiorni *et al*. 46 have screened 12 miRNAs for their functionality in modulating CFTR expression levels. As a result, two miRNAs, miR-145 and miR-494, suppressed CFTR expression by directly targeting discrete sites in the 3′-UTR of CFTR. This result agreed with those of several other studies 46–48. After *in silico* identification of a list of putative miRNAs that can target CFTR mRNA, *in vitro* analysis showed that miR-101 and miR-494 can markedly suppress CFTR expression either alone or in combination 45. miRNA-based diagnostic and therapeutic applications represent an exciting possibility for fibrosis. Quantification of selected miRNAs may be used as a sensitive biomarker tool for fibrosis severity and functional suppression of CFTR-targeting miRNAs and miR-101 can be applied as a strategy to efficiently restore CFTR synthesis in patients carrying mutations that lead to insufficient protein expression.

## miRNA and TGF-β1 pathway in CP

Cystic fibrosis transmembrane conductance regulator and TGF-β1 demonstrate inseparable relations in modulating the disease progression to fibrosis 49. Transforming growth factor-β1, *via* TGF-β1 receptor I and possibly p38 Mitogen activated protein kinase (MAPK) signalling, reduces CFTR expression to impair CFTR-mediated anion secretion. This event will likely compound the effects associated with mild CFTR mutations and ultimately compromise CFTR protein function 49. Therefore, targeting the miRNAs implicated in TGF-β1 formation will not only restore the normal function of CFTR but also directly reduce the possibility of fibrosis development (Fig.1).

During embryonic development, human cells are organized into a complex network of tissues, organs and organ systems that perform the normal physiological processes of life. One of the most ubiquitous regulators of embryonic development and physiological and cellular processes is the TGF-β superfamily of cytokines. Excessive TGF-β signalling has long been implicated in the pathogenesis of fibrotic diseases through the process of deposition of ECM 49. Transforming growth factor-β signalling through Mothers against decapentaplegic homolog 3 (SMAD3) directly promotes expression of type 1 collagen, a major component of ECM 50. SMAD3 plays a critical role in TGF-β induced fibrosis in several organ systems. Another important effector of TGF-β-induced fibrosis is connective tissue growth factor, which further promotes collagen synthesis and myofibroblast differentiation 49. As a result, epithelial cells undergo conversion into mesenchymal cells, which are associated with increased expression of fibroblast-associated proteins, such as alpha smooth muscle actin (SMA).

**Figure 2:**
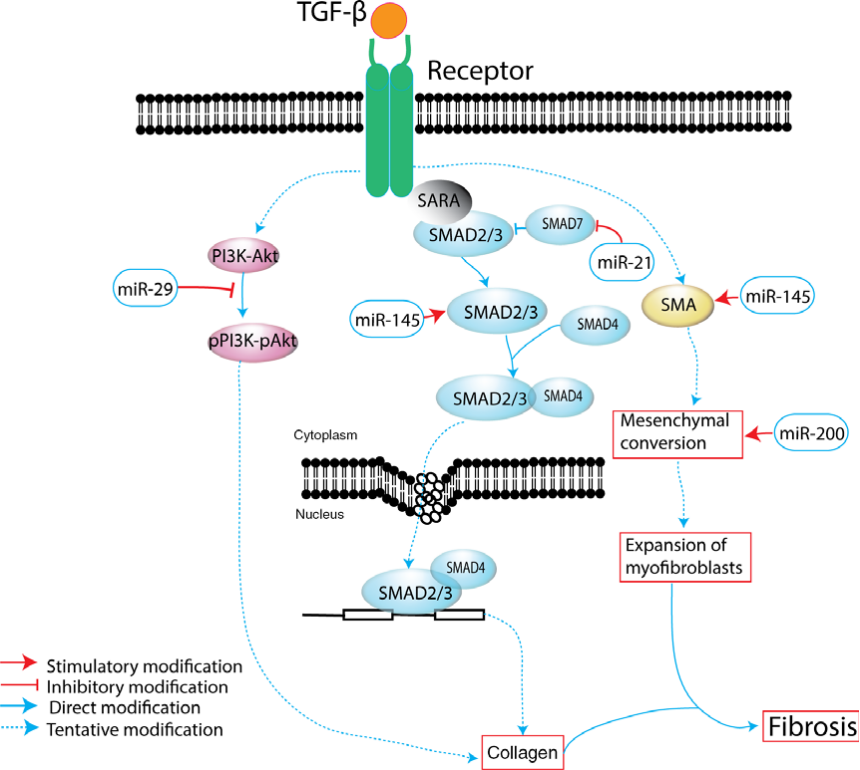
Pathogenesis of TGF-β1-induced fibrosis and miRNAs implicated in disease progression.

Transforming growth factor-β1 is centrally important in wound healing, fibrosis and negative regulation of inflammation 49. Transforming growth factor-β1 directs key cellular processes, including proliferation, survival, differentiation, motility and adhesion. Precise control of TGF-β1 expression is required for normal embryogenesis. Dysregulated TGF-β1 synthesis is an important component of carcinogenesis and metastasis 51. Increased expression of TGF-β1 is a key driver of scarring in most human diseases typified by fibrogenesis. Furthermore, miRNA is involved in TGF-β1-mediated membrane type-1-MMP (MT1-MMP) expression in pancreatic cancer 52. As a common pathological feature of CP, TGF-β1 activates PSCs, leading to pancreatic fibrosis 53. Given that the PSCs leading to fibrosis are activated in the pancreas through TGF-β1, the repression of TGF-β1 expression by miRNA represents a potential target therapy. Transforming growth factor-β1-associated downregulation of miRNAs involves the miR-29 family, miR-200 family and miR-21 (Fig.2).

### miR-29

The miR-29 family consists of three members encoded from two distinct genomic loci. In humans, a bicistronic transcript from chromosome 7q32.3 yields miR-29b-1 and miR-29a, whereas miR-29b-2 and miR-29c are derived from a bicistronic transcript from chromosome 1q32.2. All members exhibit an identical seed sequence that allows targeting of the same genes. The miR-29s are widely expressed with the most prominent expression in the kidneys, lungs and heart 54. The most well-documented function of miR-29 is its role in the prevention of tissue fibrosis. miR-29 is downregulated in the fibrotic border zone of myocardial infarction, in the lungs of idiopathic pulmonary fibrosis (IPF) patients and in the skin fibroblasts of systemic sclerosis patients 55. Expression of miR-29 is also decreased in mouse models of renal fibrosis 56,57, and the delivery of miR-29 into the kidney by ultrasound-microbubble-mediated gene transfer technique attenuates renal fibrosis in these mice 58. Several studies have implicated TGF-β signalling in the downregulation of miR-29.

Transforming growth factor-β1 promotes the expression of collagen, especially the type 1 and alpha 1 collagen *via* PI3k-Akt pathway, thus leading to fibrosis. However, miR-29 blocks this activation pathway 55. Moreover, miR-29 occupies an important role in integrating functionally connected pathways involved in fibrogenesis 58. Thus, elevated expression of miR-29 may play an important role in the pathogenesis of diseases related to fibrogenic reactions in human lung fibroblasts. These results also highly suggest that specific inhibitors of the PI3K-Akt cascade may demonstrate beneficial effects in preventing pathogenic fibrosis in human lung fibroblasts. Furthermore, miR-29a and miR-29b contribute to pancreatic beta-cell-specific silencing 59. Secondary diabetes occurring in CP subsequent to destruction of pancreatic β-cells is distinct because this condition involves β-cell dysfunction amidst an inflammatory milieu 29. Additionally, miR-29 may contribute to this molecular event in CP.

### miR-200

The miR-200 family consists of five members, namely, miR-200a, miR-200b, miR-200c, miR-429 and miR-141. In humans, a polycistronic transcript from chromosome 1 yields miR-200a, miR-200b and miR-429, whereas miR-200c and miR-141 are derived from a bicistronic transcript from chromosome 12 60. On the basis of their seed sequence, the family members can be classified into two functional groups. Group 1 consists of miR-200b, miR-200c and miR-429, whereas group 2 comprised miR-200a and miR-141. The differences in seed sequence of the two groups result in different mRNA targets. miR-200 is enriched in the kidneys and lungs, where it functions to maintain epithelial differentiation. miR-200 has recently been implicated in tissue fibrosis. The expression of miR-200 is decreased in a mouse model of lung fibrosis, and the delivery of miR-200c has reduced fibrosis 60. Despite the already elevated level of miR-200s, delivery of miR-200b-precursor by the renal press-mediated transfection method to further increase miR-200 expression has ameliorated fibrosis in mice 61,62.

The mechanism for the antifibrotic effects of miR-200 may involve prevention of epithelial-mesenchymal transition (EMT) 62 and TGF-β signalling through activation of mesenchymal transcription factors, such as ZEB1 and ZEB2, which are potent inducers of EMT. Interactions between miR-200 and ZEB1and ZEB2 are confirmed in pancreatic cancer 63. Conversely, *in vitro* assays demonstrate that miR-200s prevent TGF-β-mediated EMT through repression of ZEB1 and ZEB2 64. Furthermore, miR-200s are predicted to repress TGF-β2, which may inhibit EMT and prevent fibrosis 65. Additionally, the miR-200 family regulates the expression of MT1-MMP, and phosphatase and tensin homolog leads to aggressive behaviour of pancreatic tumour cells 66. These data suggest miR-200 as a potential biomarker or therapeutic target of CP management.

### miR-21

In humans, miR-21 maps to chromosome 17q23.2, in which this protein overlaps with the protein-coding gene TMEM49. The normal function of miR-21 is to limit injury and aid in tissue repair. Paradoxically, miR-21 is a recently identified, typical miRNA functioning as a regulator involved in apoptosis, as well as inflammatory and fibrotic signalling pathways 67. Moreover, miR-21 is among the most highly up-regulated miRNAs during tissue injury, such as acute pancreatic injury. Persistent expression of miR-21 perturbs tissue repair and contributes to tissue and chronic fibroses. Similarly, miR-21 significantly increased in activated HSCs 68. Furthermore, miR-21 is up-regulated in cardiac fibroblasts in failing mouse and human hearts, and anti-miR-mediated inhibition of miR-21 through the jugular vein catheter attenuates fibrosis and improves cardiac function in mouse models of heart failure 69. The antifibrotic effects of miR-21 inhibitors are also observed in the lungs and kidneys 69. miR-21 is up-regulated in the lungs of IPF patients, and inhibition of miR-21 by intratracheal instillation of antisense attenuates fibrosis in mouse models of lung fibrosis 70. Thus, inhibition of miR-21 is a novel therapeutic strategy for progressive tissue fibrosis, and miR-21 may be a critical therapeutic target for renal fibrosis 71.

Similar to miR-200, miR-21 is involved with the EMT process during onset of fibrosis 72. In fact, miR-21 has been up-regulated with stimulation by TGF-β1 68,73. Transforming growth factor-β signalling promotes miR-21 synthesis not only by increasing transcription but also by enhancing post-transcriptional processing of pri-miR-21 73. Consequently, miR-21 promotes TGF-β signalling. Thus, miR-21 functions in a feed-forward loop leading to TGF-β signal amplification. Overexpression of miR-21 in fibroblasts decreased the levels of SMAD7, whereas knockdown of miR-21 increased its expression. SMAD7 may also be the target of miR-21 74. miR-21 may function in an amplifying circuit to enhance TGF-β signalling events. miR-21 also promotes fibrosis through other mechanisms, such as activation of ERK/MAP kinase signalling, inhibition of apoptosis and promotion of proliferation of fibroblasts 69. Indeed, miR-21 is induced early in pancreatic ductal adenocarcinoma precursor lesions 75. We speculate that the overexpression of miR-21 during tissue injury enhances TGF-β signalling events and leads to pancreatic fibrosis. Thus, miR-21 may play a key pathogenic role in CP.

### miR-145

SMAD3, a key element of the TGF-β1 inflammatory pathway, was identified as a target of miR-145 76. An inverse correlation was evident between the expression of miR-145 and SMAD3 in fibrosis patients: miR-145 was increased in nasal airway cells of cystic fibrosis *versus* non-cystic fibrosis individuals 76. Yang *et al*. 77 indicated that miR-145 also enhances the contractility of lung fibroblasts and contributes to fibrosis. miR-145 promotes latent TGF-β1 activation in a delayed manner, as suggested by the elevated expression of fibronectin in lung fibroblasts, 7 days after they were transfected with miR-145. This increased activity of latent TGF-β1 is most likely attributed to the enhanced SMA expression and the resulting increased myofibroblast contractility. Similarly, the association between miR-145 and fibroblast differentiation toward myofibroblasts is demonstrated in cardiac myofibroblast differentiation 78. Additionally, miR-145 plays a critical role as a regulator of Wnt/β-catenin activity in response to TGF-β and hypoxia 79. A recent study confirmed that miR-145 was differentially expressed during PSC activation 80. Thus, miR-145 is also a promising regulator during TGF-β activation of PSCs 80.

## Designing miRNA as an effective diagnostic or therapeutic tool

The basic understanding of miRNA biology has led to the development of novel approaches to manipulate miRNA expression and function. The approaches use synthetic oligonucleotides that either inhibit or enhance the expression of specific miRNAs. The inhibitors called anti-miRNAs contain a sequence complementary to a mature miRNA of interest. Thus, the anti-miRs bind and sequester specific miRNAs and relieve the repression of the target mRNAs. Conversely, miRNA-mimics are duplex oligonucleotides with a sequence identical to a mature miRNA of interest. Once inside the cell, the miRNA-mimics associate with the miRISC complex and post-transcriptionally inhibit the target mRNAs. Anti-miRs possess the characteristics of an ideal therapeutic agent 81. Moreover, they represent novel tools to study the biological roles of specific miRNAs *in vitro* and *in vivo*. Therefore, designing the specific anti-miRNAs targeting specific pathways stands for a potential therapy at the genetic level to modulate the expression levels of various proteins. The discovery of miRNA inhibitors and enhancers greatly pushed forward the application of miRNAs as therapies in CP. Specific oligonucleotides may be designed to modulate miRNA levels, thus ultimately regulating protein expression levels. However, a personalized miRNA therapy is very important because a single miRNA has multiple targets. To maintain other functions without disturbance, a specific tissue should be targeted under certain conditions.

Interestingly, when a miRNA is diluted at a concentration lower than the affinity for their targets, the overall effect of the miRNA on gene expression is expected to be negligible 82. Thus, in this case, significant regulation of miRNA activity through competitive transcripts is theoretically possible but only if the overall abundance of the competitor is much higher than the total number of miRNAs. This finding offers another option in designing miRNA-related tools to regulate mRNA expression. This option can be considered as a more ‘natural’ way in modulating *in vivo* miRNA because no synthetic oligonucleotides have been introduced with potentially less prone to side effects.

Apart from the potential role in developing suitable and more efficient therapies, miRNAs also represent as potential diagnostic tools. Recent studies on circulating miRNAs have generated an alternative approach to identify minimally invasive biomarkers for different diseases, among which hepatitis is a further studied one 83. The aberrant levels of a vast diversity of miRNAs in serum are frequently correlated with certain diseases. With the advancement of detection methods, screening for the aberrant presence of miRNAs becomes easier and more convenient. Therefore, the correlations between indicative miRNAs and disease progression should be established to provide early-stage disease diagnosis and apply preventive measures. However, various miRNAs are abnormally expressed at the disease condition of CP; thus, to avoid misinterpretation, a detailed miRNA profile should be created and combined with other diagnostic methods.

Although miRNA stands for a novel and potential therapeutic target and therapeutic silencing of miRNAs facilitates development of novel drugs, a major drawback of exploiting miRNAs as therapies is the possibility of adverse side effects because of the multiple drug targets for a single miRNA. The biological properties of miRNAs, in which a single miRNA binds to multiple targets and regulate various signalling pathways simultaneously, will potentially offset the desired therapeutic effects. Moreover, although many experimental studies have confirmed the capacity of miRNAs or antagomirs to detect or treat fibrosis-related diseases 54,57,60,77,84, adequate evaluation of their accuracy, efficacy, and cost-effectiveness is required.

## Summary

In the current review, we have discussed miRNA as a potential diagnostic and therapeutic target for the treatment of CP fibrosis. Although some miRNA families have been discovered to demonstrate a direct link to tissue fibrosis, such as miR-21, miR-29, and miR-200, other unknown miRNA families play important roles in modulating genetic expression that is indirectly related to CP progression. The specific miRNAs involved in the disease progression remain to be investigated. Examples are the miRNAs involved in CFTR mutation, alcohol consumption, as well as the control of ROS. Identifying these specific miRNA families can be extremely important to better identify CP at an early stage and correct fibrosis with disease progression.

A major challenge for CP is the identification of clinically effective treatments and biomarkers for the diagnosis, prognosis and treatment efficacy. Knowledge regarding miRNA in human pancreatic diseases may eventually lead to serum or tissue-specific biomarkers with clinical utility. However, prior to clinical application, major challenges, such as the need for careful validation of diagnostic miRNA candidates in well-annotated clinical trials and the technical issues involved, such as quantification, standardization, and normalization of expression, are faced in related research.

As a novel frontier, applying miRNAs in treatments have been explored in hepatitis. The very first anti-miRNA oligonucleotide, miravirsen, has been tested in clinical trials and has shown promising results as a therapeutic agent against chronic hepatitis C virus infection 85. However, investigations on applying miRNA or anti-miRs in treatments of CP remain at the development stage, and further research efforts are needed. The encouraging progress has set a clinical model for further research on CP with miRNA treatments. The rapid progress in therapeutic interventions using miRNA-based strategies for CP will facilitate more novel approaches that can build on the existing and emerging knowledge regarding miRNAs.

## Funding

This study was supported by the National Natural Science Foundation of China (grant nos. 81100316, 81100256, 81470883 and 81300355), and of Scientific Research Foundation for Young Talents of Changhai Hospital (grant nos. CH201304 and CH201515).

## Conflicts of interest

No conflicts of interest exist in this study.
